# An Unnoticed Symptom of Ipecacuanha, Clinically Verified by Dr. Mossa

**Published:** 1889-05

**Authors:** 


### AN UNNOTICED SYMPTOM OF IPECACUANHA CLINICALLY VERIFIED BY DR. MOSSA, STUTTGART.

(*Allg. Hom. Zeitung*, No. 3, 1889.)

A young man suffered from a queer toothache—stitching pains in right cheek, radiating from the various teeth of the upper maxilla into the temples, ears, and nose, < at night. As Mercury failed, he had the offender extracted, but no relief followed, and he complains now, off and on, in the upper teeth, of a *painful wrench as if the teeth were pulled out*, especially in daytime, and it did not trouble him much at night. He received Ipec.$^{3d}$, three times a day, and a few doses cured him.

In Hahnemann's *Materia Medica Pura*, Engl. ed. 788, S. 41 (G. E. third v. 175) we read: "A pain in the teeth, as if they were pulled out, in fits (after eight hours); very violent pain in a hollow tooth when biting, immediately, as if it was pulled out, causing loud howling and crying out and thereafter constant tearing in it. In the first edition Hahnemann puts the symptom in ( ), as if doubtful, but Weber gives it in full and open. Hering mentions in his *Domestic Physician:* Arnica, Causticum, Nux-mosch., Nux-v., Phos-ac., Rhus for pulling, tearing pains, a symptom which we also meet in Coccinella, Cyclamen, Manganese, Mezereum, and the north-pole of the magnet. Raue does not mention Ipecacuanha, and we fail to read of it as a remedy for toothache in Burr, Jousaet, or Kafka. It may be, therefore, of interest to give a hint as to its use to the students of materia medica.

